# Heterologous production of active form of beta-lytic protease by *Bacillus subtilis* and improvement of staphylolytic activity by protein engineering

**DOI:** 10.1186/s12934-021-01724-x

**Published:** 2021-12-28

**Authors:** Takahiro Hioki, Daichi Yamashita, Masatoshi Tohata, Keiji Endo, Akihito Kawahara, Mitsuyoshi Okuda

**Affiliations:** 1 Biological Science Research, Kao Corporation, 1334 Minato, Wakayama, Wakayama 640-8580 Japan; 2 Biological Science Research, Kao Corporation, Haga , Tochigi 2606 Akabane, Ichikai321-3497 Japan; 3 Safety Science Research, Kao Corporation, 2606 Akabane, Ichikai, Haga, Tochigi 321-3497 Japan

**Keywords:** *Bacillus subtilis*, Heterologous expression, Protein engineering, *Staphylococcus aureus*, MRSA, Beta-lytic protease, BLP, M23 protease family

## Abstract

**Background:**

Most of the proteases classified into the M23 family in the MEROPS database exhibit staphylolytic activity and have potential as antibacterial agents. The M23 family is further classified into two subfamilies, M23A and M23B. Proteases of the M23A subfamily are thought to lack the capacity for self-maturation by auto-processing of a propeptide, which has been a challenge in heterologous production and application research. In this study, we investigated the heterologous expression, in *Bacillus subtilis*, of the *Lysobacter enzymogenes* beta-lytic protease (BLP), a member of the M23A subfamily.

**Results:**

We found that *B. subtilis* can produce BLP in its active form. Two points were shown to be important for the production of BLP in *B. subtilis*. The first was that the extracellular proteases produced by the *B. subtilis* host are essential for BLP maturation. When the host strain was deficient in nine extracellular proteases, pro-BLP accumulated in the supernatant. This observation suggested that BLP lacks the capacity for self-maturation and that some protease from *B. subtilis* contributes to the cleavage of the propeptide of BLP. The second point was that the thiol-disulfide oxidoreductases BdbDC of the *B. subtilis* host are required for efficient secretory production of BLP. We infer that intramolecular disulfide bonds play an important role in the formation of the correct BLP conformation during secretion. We also achieved efficient protein engineering of BLP by utilizing the secretory expression system in *B. subtilis*. Saturation mutagenesis of Gln116 resulted in a Q116H mutant with enhanced staphylolytic activity. The minimum bactericidal concentration (MBC) of the wild-type BLP and the Q116H mutant against Staphylococcus aureus NCTC8325 was 0.75 μg/mL and 0.375 μg/mL, respectively, and the MBC against Staphylococcus aureus ATCC43300 was 6 μg/mL and 3 μg/mL, respectively.

**Conclusions:**

In this study, we succeeded in the secretory production of BLP in *B. subtilis*. To our knowledge, this work is the first report of the successful heterologous production of BLP in its active form, which opens up the possibility of industrial use of BLP. In addition, this study proposes a new strategy of using the extracellular proteases of *B. subtilis* for the maturation of heterologous proteins.

**Supplementary Information:**

The online version contains supplementary material available at 10.1186/s12934-021-01724-x.

## Background

The M23 protease family in the MEROPS database is a family of zinc-dependent metallopeptidases with a zinc-binding HXH motif [1]. Many of the enzymes in this family have glycylglycine endopeptidase activity, which cleaves peptide linkers that cross-link cell wall peptidoglycans to lyse Gram-positive bacteria such as staphylococci [2]. Since these enzymes have the activity to kill pathogenic *Staphylococcus aureus*, which has a pentaglycine linker in its cell wall peptidoglycan, these proteins are expected to find application as antimicrobial agents in medicine, veterinary science, and the food industry [3]. These enzymes also are effective against antimicrobial-resistant *S. aureus*, and therefore are being considered as alternatives to, or for use in combination with, existing antimicrobial agents [4, 5].

The M23 family is further classified into two subfamilies, M23A and M23B, based on amino acid sequence homology [1]. The M23A subfamily includes beta-lytic protease (BLP) from *Lysobacter enzymogenes* [6] and LasA protease (staphylolysin) from *Pseudomonas aeruginosa* [7], while the M23B subfamily includes lysostaphin from *Staphylococcus simulans* [8] and ALE-1 from *Staphylococcus capitis* [9]. Among the M23 family members, lysostaphin in particular has been extensively studied for application as an antimicrobial agent; this enzyme has been shown to be effective in a number of preclinical animal models and to show efficacy in a small number of clinical trials [10]. On the other hand, there are few reports of applied research on M23A subfamily enzymes, with only a few papers describing the efficacy of LasA protease in preclinical animal models [5, 11]. Because LasA protease has broader substrate specificity than lysostaphin [12, 13], it may be effective in the treatment of opportunistic infections caused by staphylococci other than *S. aureus* [3].

A BLP belonging to the M23A subfamily was first identified in *L. enzymogenes*, [6] and BLPs with almost identical mature sequences have been identified in *Achromobacter lyticus* M497-1 [14], *Lysobacter* sp. IB-9374 [15], and *Lysobacter capsici* VKM B-2533T [16]. BLP has broader substrate specificity than lysostaphin and shows lytic activity not only against *S. aureus*, including methicillin-resistant *S. aureus* (MRSA), but also against other staphylococci and *Micrococcus luteus* [15, 16]. Although *M. luteus* does not have a glycylglycine in the peptide linker of its cell wall peptidoglycan and is resistant to lysostaphin and LasA protease [12], BLP shows activity in degrading the l-Ala-d-Ala bond of the *M. luteus* peptide linker [17]. Several studies have proposed the application of BLPs as antimicrobial agents [16, 17], but (to our knowledge) no application studies of BLPs have been reported to date.

**Fig. 1:**
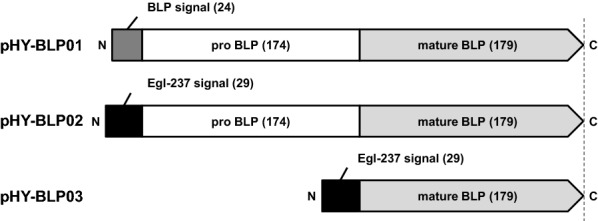
Schematic diagram of the ORF region of the expression vectors of BLP. In the figure, “N” and “C” indicate the N-terminus and C-terminus of the protein, respectively, and the number of the amino acid residues in each sequence is given in parentheses. The open reading frame (ORF) of pHY-BLP01 encodes the full-length beta-lytic protease (BLP) protein, including the signal, pro, and mature sequence. The ORF of pHY-BLP02 encodes the pro and mature sequence of BLP fused to the signal sequence of Egl-237. The ORF of pHY-BLP03 encodes the mature sequence of BLP fused to the signal sequence of Egl-237. Each ORF was placed downstream of the *egl-237* promoter and cloned into pHY300PLK

One of the reasons for the lack of applied research on M23A subfamily proteases has been the lack of available heterologous expression systems [3]. The M23A proteases have a pre-pro peptide at the N-terminus of the mature region (Fig. 1). In *P. aeruginosa*, LasA protease is secreted as a pro-protein and is matured through processing by other proteases [18]. When expressed heterologously in *Escherichia coli*, LasA protease was produced as the pro-protein with no activity [19]. Heterologous expression of BLP derived from *L. capsici* VKM B-2533T in *E. coli* resulted in accumulation of the pro-protein in inclusion bodies [16]. Pseudoalterin, an M23A protease from *Pseudoalteromonas* sp. CF6-2, also lacks the ability to self-mature, precluding the heterologous production of the active enzyme in *E. coli* [20]. The only report of successful heterologous production of the active form of M23A protease is the expression of pseudoalterin using *Pseudoalteromonas* sp. SM20429 as a host [21]. The enzyme from *Pseudoalteromonas*, which cannot be matured in *E. coli*, can be produced as the mature protein by using a host of the same genus. As described above, the lack of self-maturation ability is a major issue in the heterologous production of M23A subfamily proteases. Heterologous production of M23A subfamily proteases in an active form in a high-level protein-producing host is essential for industrial applications.

*Bacillus subtilis* is a Gram-positive, non-pathogenic bacterium that is widely used as a host for heterologous protein production because of its excellent ability to secrete proteins into the medium [22]. The secretory production of target proteins into the medium can simplify downstream processing in industrial production compared to intracellular production, which requires cell disruption. In the expression of heterologous proteins in *B. subtilis*, degradation of the product by endogenous extracellular proteases is often a bottleneck. *B. subtilis* releases at least nine different proteases into the culture supernatant, and there are many reports that multiple disruptions of the corresponding genes can improve heterologous protein productivity [23, 24].

In the present study, we investigated the heterologous expression of the BLP by *B. subtilis*. Unexpectedly, secretory expression in *B. subtilis* resulted in the accumulation of the mature form of the BLP in the supernatant. To our knowledge, this work is the first report of successful heterologous production of BLP in its active form. We also employed site-directed mutagenesis to improve the staphylolytic activity of BLP using the *B. subtilis* expression system.

## Results

### Secretory expression of BLP by *B. subtilis*

**Fig. 2:**
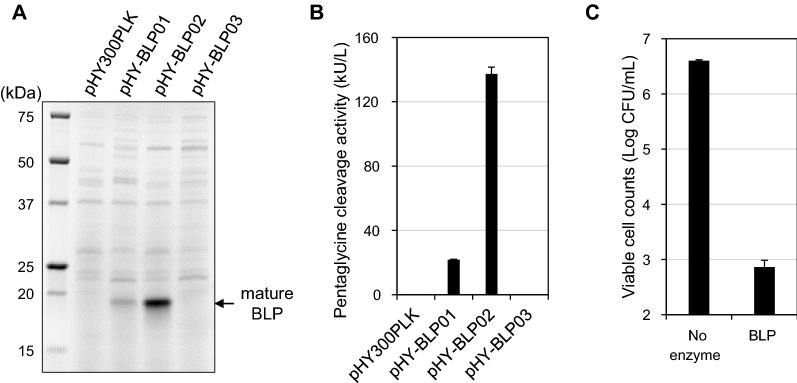
Secretion and activation of BLP by *B. subtilis. B. subtilis* strain 168 was transformed with pHY300PLK (empty vector), pHY-BLP01, pHY-BLP02, or pHY-BLP03 for BLP expression. Cells were grown at 30 °C for 72 h in modified 2 × L-Mal medium. **A** SDS-PAGE analysis of culture supernatants. The supernatants were diluted two-fold with SDS reducing buffer. Five microliters of each sample were applied to SDS-PAGE. The expression plasmids are indicated above the image. The arrow indicates the position of mature BLP. **B** Pentaglycine cleavage activity assay of culture supernatants using the pentaglycine-containing FRET substrate FRET-GGGGG. **C** Bactericidal assay of the purified BLP against *S. aureus* NCTC8325. The bactericidal activity was measured at a final protein concentration of 1.5 µg/mL. The results presented are the means of three individual experiments. Error bars represent the standard errors of the means

We investigated the possibility of heterologous secretory expression of BLP (GenBank: BAV99603.1) derived from *L. enzymogenes* M497-1 (formerly *Achromobacter lyticus*) [25] using *B. subtilis* strain 168 as a host. For secretory expression in *B. subtilis*, it is necessary to add a secretory signal peptide at the N-terminus of the target protein. In the present study, we used the native signal sequence of BLP and the signal sequence of Egl-237 (GenBank accession number: BAB19360.1), which is known to have a high secretion efficiency [26]. Three types of expression plasmids were used: one encoding a full length BLP, including the signal, pro, and mature sequence (pHY-BLP01); a second encoding the pro and mature sequence of BLP fused to the signal sequence of Egl-237 (pHY-BLP02); and a third encoding the mature sequence of BLP fused to the signal sequence of Egl-237 (pHY-BLP03) (Fig. 1). Evaluation by sodium dodecyl sulfate–polyacrylamide gel electrophoresis (SDS-PAGE) revealed the presence of 19-kDa bands corresponding to the mature form of BLP in the culture supernatants of cells harboring pHY-BLP01 and pHY-BLP02 (Fig. 2A). A pentaglycine cleavage activity assay using the pentaglycine-containing Förster (Fluorescence) Resonance Energy Transfer (FRET) substrate FRET-GGGGG detected activity in the culture supernatant of cells harboring pHY-BLP01 and pHY-BLP02 (Fig. 2B). Cells harboring the pHY-BLP02 plasmid, which encodes BLP with the Egl-237 secretion signal, produced the mature BLP at 0.8 g/L, a productivity that exceeded that seen in cells harboring pHY-BLP01, which encodes BLP with the original secretion signal. Neither a band corresponding to the mature form of BLP nor the pentaglycine cleavage activity was detected in the culture supernatant of cells harboring pHY-BLP03, indicating that the propeptide was essential for the secretory expression of BLP by *B. subtilis*. BLP was purified from the culture supernatant of cells harboring pHY-BLP02 by use of a cation exchange column and subjected to N-terminal amino acid sequencing. The N-terminal sequence of the BLP produced by pHY-BLP02-bearing *B. subtilis* was SPNGL, which was the same as that of the mature BLP in Achromopeptidase, a commercial lytic enzyme produced by *L. enzymogenes* M497-1 [14]. Furthermore, the BLP produced by *B. subtilis* showed bactericidal activity against *S. aureus*. Incubation of S. aureus NCTC8325 cells with the purified BLP at 1.5 µg/mL at 30 °C for 60 min reduced log CFU/mL from 6.6 to 2.9 compared to no enzyme (Fig. 2C). Thus, we demonstrated that BLP can be produced as the mature, active form using *B. subtilis* heterologous secretory expression system.

### Effect of *B. subtilis* extracellular proteases on the maturation of BLP

**Fig. 3:**
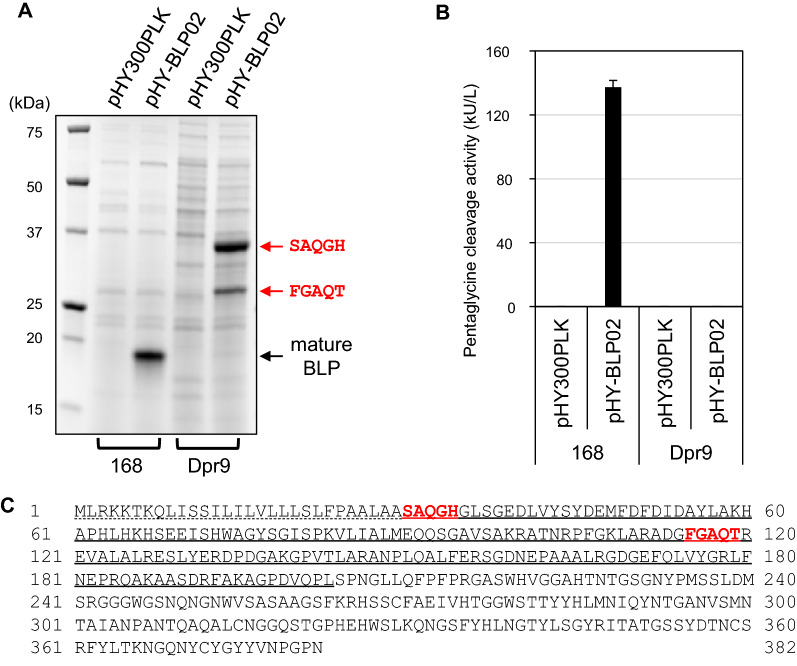
Maturation of BLP by extracellular proteases of *B. subtilis.* The *B. subtilis* Dpr9 strain and the parental strain 168 were transformed with pHY300PLK (empty vector) or pHY-BLP02. Cells were grown at 30 °C for 72 h in modified 2 × L-Mal medium. **A** SDS-PAGE analysis of culture supernatants. The supernatants were diluted two-fold with SDS reducing buffer. Five microliters of each sample were applied to SDS-PAGE. The expression plasmids and hosts are indicated above and below the image, respectively. The black arrow indicates the position of mature BLP. The red arrows indicate the positions of unprocessed or partially cleaved forms of pro-BLP, and their N-terminal sequences are shown. **B** Pentaglycine cleavage activity assay of culture supernatants using the pentaglycine-containing FRET substrate FRET-GGGGG. The results presented are the means of three individual experiments. Error bars represent the standard errors of the means. **C** Amino acid sequence of BLP encoded by pHY-BLP02. The signal sequence is underlined with a dotted line, and the pro sequences is underlined with a straight line. The N-terminal sequences detected in the culture supernatant of Dpr9 strain are shown in red bold

Some proteases with propeptides have the ability to self-mature, while others do not. Subtilisin E, an alkaline serine protease produced by *B. subtilis* 168, matures by cleaving the propeptide through its own protease activity after folding [27]. In contrast, LasA protease and pseudoalterin, which are classified in the same M23A subfamily as BLP, do not show self-maturation ability [18, 20]. Since BLP also may lack the ability to self-mature, we tested whether proteases from *B. subtilis* contribute to the maturation of BLP. *B. subtilis* strain Dpr9, in which nine genes encoding extracellular proteases have been deleted, was cultured following transformation with pHY-BLP02. The protease deficiency resulted in the disappearance of the pentaglycine cleavage activity and of the band corresponding to mature BLP in the supernatant, while two additional bands at higher molecular weight, ~ 28 kDa and 36 kDa, were observed (Fig. 3). The N-terminal sequence of the 28 kDa band was identified as FGAQT, which is consistent with the sequence of residues 86–90 of the propeptide of BLP. The N-terminal sequence of the 36 kDa band was identified as SAQGH, which is consistent with the N-terminal of the propeptide of BLP. These results indicated that BLP does not self-mature into the active form in the culture supernatant of *B. subtilis*, and that the extracellular proteases of *B. subtilis* are essential for the maturation of BLP.

### Importance of intramolecular disulfide bonds in BLP

**Fig. 4:**
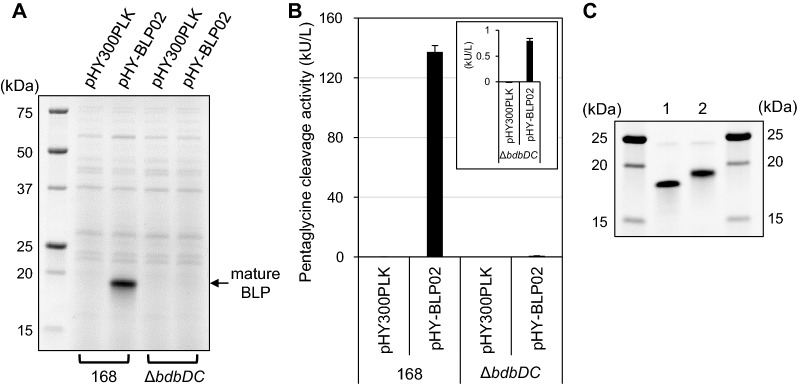
Formation of intramolecular disulfide bond of BLP. The *B. subtilis* Δ*bdbDC* strain and the parental strain 168 were transformed with pHY300PLK (empty vector) or pHY-BLP02. Cells were grown at 30 °C for 72 h in modified 2 × L-Mal medium. **A** SDS-PAGE analysis of culture supernatants. The supernatants were diluted two-fold with SDS reducing buffer. Five microliters of each sample were applied to SDS-PAGE. The expression plasmids and hosts are indicated above and below the image, respectively. The arrow indicates the position of mature BLP. **B** Pentaglycine cleavage activity assay of culture supernatants using the pentaglycine-containing FRET substrate FRET-GGGGG. Since the activity detected in the Δ*bdbDC* strain was very low, it was also included in the inset. The results presented are the means of three individual experiments. Error bars represent the standard errors of the means. **C** Non-reducing and reducing SDS-PAGE analysis of the purified BLP. The purified BLP was applied to SDS-PAGE at 500 ng/lane under non-reducing (lane 1) or reducing (lane 2) conditions

BLP is thought to contain two intramolecular disulfide bonds, one each between cysteine residues 66 and 112 and between cysteine residues 156 and 169 [28]. In the heterologous secretory expression in *B. subtilis* of the *E. coli* alkaline phosphatase PhoA, which contains intramolecular disulfide bonds, the *bdbDC* operon, which encodes thiol-disulfide oxidoreductases, plays an important role in the formation of the disulfide bonds [29, 30]. *B. subtilis* strain Δ*bdbDC*, which lacks the *bdbDC* genes (UniProt O32217, O32218), was cultured following transformation with pHY-BLP02. No BLP bands were detected in the SDS-PAGE of the culture supernatant of the Δ*bdbDC* strain (Fig. 4A). A > 100-fold decreased level of pentaglycine cleavage activity was detected in the culture supernatant of the Δ*bdbDC* strain compared to strain 168 (Fig. 4B). These results indicated that the *bdbDC* operon plays a pivotal role in the secretion of BLP by *B. subtilis*. Non-reducing and reducing SDS-PAGE analysis of the BLP secreted by *B. subtilis* 168 (*bdbDC*^+^) revealed that this protease exhibits faster mobility in the non-reducing condition than in the reducing condition (Fig. 4C). This result suggested that the BLP secreted by *B. subtilis* 168 has one or more intramolecular disulfide bonds.

### Improvement of staphylolytic activity of BLP by protein engineering

**Fig. 5:**
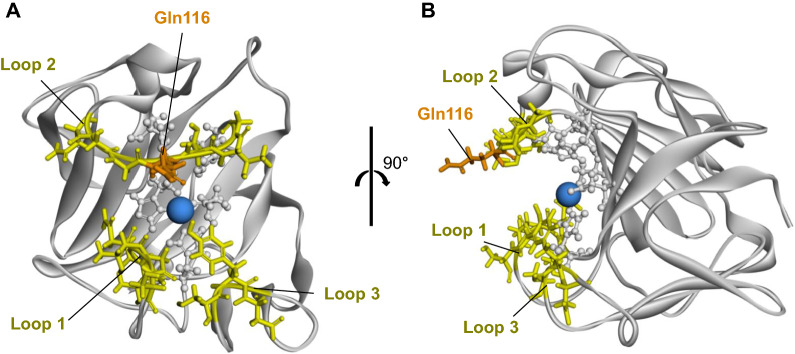
Homology modeling of mature BLP. Ribbon diagram of a predicted BLP structure. The catalytic residues (His22, Asp36, His82, His121, His123) are shown in ball-and-stick format. The zinc ion is shown as a blue ball. Loops 1 to 3, which form the entrance to the catalytic cleft, are shown in yellow in stick format (Loop 1: residues 23–27, Loop 2: residues 113–119, and Loop 3: residues 151–153). Gln116 is shown in orange. The BLP structure was predicted using Discovery Studio (Dassault Systèmes). The template was LasA protease (PDB code 3IT5), which shares 43% amino acid identity with mature BLP. **A** Cleft side. **B** Rotated 90° from (**A**)

**Fig. 6:**
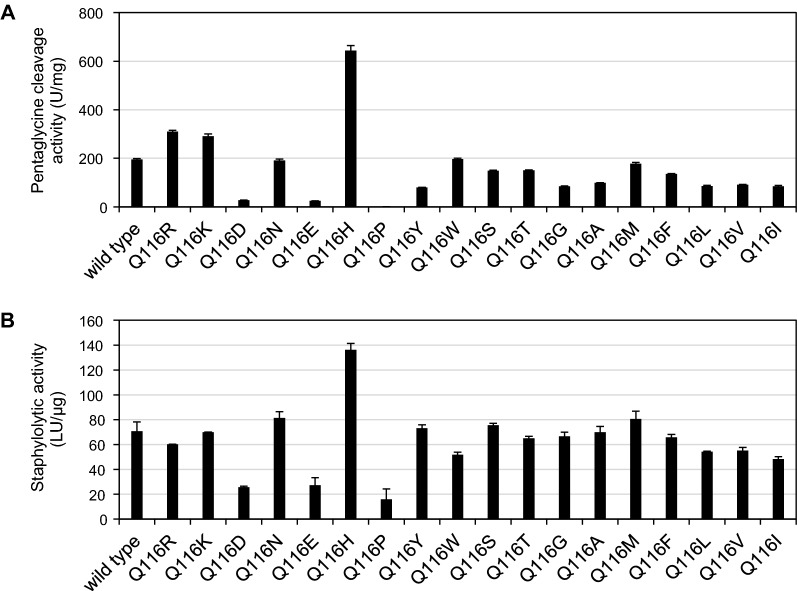
Specific activity of Gln116 saturation mutants of BLP. **A** Pentaglycine cleavage activity assay of BLP variants using the pentaglycine-containing FRET substrate FRET-GGGGG. **B** Staphylolytic activity assay of BLP variants. The results presented are the means of three individual experiments. Error bars represent the standard errors of the means

**Fig. 7:**
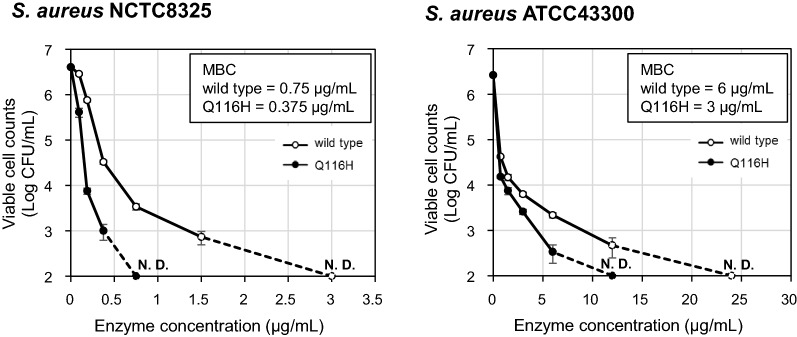
MBC of wild-type BLP and Q116H mutant against *S. aureus.* Bactericidal assay against *S. aureus* NCTC8325 and *S. aureus* ATCC43300. The enzyme concentrations at which no colonies formed are plotted on the x-axis, noted as N.D. (not detected), and connected to the curve at the next lower concentration by a dotted line. The results presented are the means of three individual experiments. Error bars represent the standard errors of the means. The minimum bactericidal concentration (MBC) of each enzyme is listed in the upper right corner of the plot area

We next sought to use protein engineering to enhance the staphylolytic activity of BLP using the *B. subtilis* heterologous secretory expression system. A structural model of mature BLP was generated using the crystal structure of LasA protease (PDB code 3IT5) as a template; notably, LasA protease shares 43% amino acid sequence identity with mature BLP (Fig. 5). The entrance to the catalytic cleft of BLP is composed of three loops (Loop 1: residues 23–27, Loop 2: residues 113–119, and Loop 3: residues 151–153). The structural model suggested that the sidechain of Gln116, which is located in Loop 2, has a large outward protruding structure, which is expected to contribute to the interaction with the substrate. Mutant *blp* genes were generated by saturation mutagenesis of the nucleotides encoding Gln116; the mutant proteins then were produced using the *B. subtilis* secretory system. Except for the Q116C mutant, which was expressed only at a low level (data not shown), 18 mutants and the wild-type BLP were expressed, purified, and evaluated. Substitution of Gln116 with basic amino acids (Arg, Lys, His) enhanced the pentaglycine cleavage activity (Fig. 6A). Among these mutant proteins, only Q116H demonstrated enhanced staphylolytic activity compared to the wild-type BLP (Fig. 6B). In contrast, substitution of Gln116 with acidic residues (Asp, Glu) or Pro greatly decreased the pentaglycine cleavage activity and the staphylolytic activity. The minimum bactericidal concentration (MBC) for the wild-type and the Q116H mutant BLPs against *S. aureus* NCTC8325 and methicillin-resistant *S. aureus* ATCC43300 were determined; the MBC against *S. aureus* NCTC8325 was 0.75 μg/mL and 0.375 μg/mL, respectively, and the MBC against *S. aureus* ATCC43300 was 6 μg/mL and 3 μg/mL, respectively (Fig. 7).

## Discussion

We found that BLP can be produced in an active form by using *B. subtilis* as a host for heterologous secretory expression. As shown in Fig. 2A, the propeptide was essential for the expression of BLP in *B. subtilis*. One of the major functions of propeptides of bacterial extracellular proteases is thought to be to assist in the folding of the mature region [31]. Lysostaphin, a member of the M23B subfamily, has a propeptide that is processed by the extracellular cysteine protease of *S. simulans*, but this propeptide is not essential for lysostaphin expression [32]. Although the pro-form of lysostaphin was active, the mature form without the propeptide was more active than the pro-form [32]. Several heterologous expression systems for lysostaphin have been developed in different hosts including *Lactococcus lactis* [33] and *Pichia pastoris* [34], and in most cases an active enzyme was produced by cloning only sequences lacking the propeptide. In contrast, in the M23A subfamily member LasA protease, mutations in the propeptide region result in a dramatic decrease in the stability and activity of the mature form, suggesting that the propeptide is important for the correct folding of the mature region [35]. In the present study, the propeptide of BLP was essential for secretory expression by *B. subtilis*, supporting the idea that the propeptide has a function in assisting the folding of the mature BLP, as is seen with LasA protease.

BLP did not show self-maturation ability in the culture medium of *B. subtilis*, and the extracellular proteases of *B. subtilis* were required for maturation. In many previous studies of protein production in *B. subtilis*, degradation of products by host extracellular proteases has been regarded as a bottleneck for heterologous protein production [23, 24]. In the case of BLP, the focus of the present study, the characteristic result was that the propeptide was cleaved by the proteases from *B. subtilis*. To our knowledge, the present work is the first demonstration that a protease from *B. subtilis* can be used for the maturation of a heterologously expressed protein. We believe that this result shows a potential novel utility for *B. subtilis* as a host for heterologous production. This observation is a useful finding, as maturation by a *B. subtilis* extracellular protease also may be of use in the expression of other heterologous proteins that do not have the ability to self-mature. In the present study, we showed that BLP does not mature in the Dpr9 strain, which is deficient in nine reported extracellular proteases (those encoded by *aprE*, *nprE*, *bpr*, *wprA*, *vpr*, *epr*, *mpr*, *nprB*, and *aprX*). We currently are trying to identify the specific protease that contributes to the maturation of BLP when expressed in *B. subtilis*. We expect that maintenance of the gene encoding that endogenous extracellular protease, while disrupting the extracellular protease genes that do not contribute to BLP maturation, will permit BLP maturation while increasing productivity due to the loss of extracellular proteases.

In the Dpr9 strain, where no BLP activity was detected in the supernatant, two novel bands were observed at approximately 36 kDa and 28 kDa. Based on the results of N-terminal sequence analysis, the band around 36 kDa is considered to be the full-length of pro-BLP (38.2 kDa), and the band around 28 kDa is considered to be a partially cleaved but inactive form of the pro-BLP (28.9 kDa) cleaved between residues Gly85 and Phe86. This partial processing could be catalyzed either by its own catalytic activity or by a host protease other than the nine that are deleted in the Dpr9 strain. During the maturation process of LasA protease in the supernatant of *P. aeruginosa* culture, a 28-kDa intermediate also has been reported; this novel band is inferred to reflect processing by the elastase and the lysine-specific protease [18]. In the process of purifying and refolding pro-BLP heterologously expressed in inclusion bodies in *E. coli*, it was reported that active BLP is obtained without the addition of any other proteases [16]. It is possible that traces of contaminating proteases in the sample contributed to the maturation of the pro-BLP.

Since the thiol-disulfide oxidoreductases BdbDC were important for BLP expression, cross-linking of the disulfide bonds might be a bottleneck for BLP production. In the secretory expression of the *E. coli* alkaline phosphatase PhoA in *B. subtilis*, suppression of the intracellular reductase and introduction of a heterologous thiol-disulfide oxidoreductase was shown to improve productivity [36]. These approaches also may be effective in BLP production.

*B. subtilis* also is a useful host when employed as a tool for protein engineering. In the present work, the secretory production of active BLP by *B. subtilis* enabled efficient protein engineering of this protease. The activity of BLP was significantly altered by the substitution of Gln116, which was predicted from the modeling structure to make a large contribution to the interaction with the substrate. In particular, the Q116H mutant showed enhanced activity (compared to the wild-type protein) in cleavage of a pentaglycine FRET substrate and lysis of *S. aureus*. The Q116H mutant showed an MBC that was two-fold lower than that of the wild-type enzyme against both *S. aureus* NCTC8325 and *S. aureus* ATCC43300 (MRSA). To our knowledge, this work is the first report of an enhanced activity mutation in the M23A protease. The cleavage activity against the pentaglycine FRET substrate correlated approximately with the bacteriolytic activity against *S. aureus*, suggesting that a simple screen using peptide substrates will be useful in seeking mutations that improve bacteriolytic activity. Further modification of the three loops that constitute the entrance to the active cleft is expected to improve the activity and change the substrate specificity of BLP.

*S. aureus* ATCC43300 (MRSA) showed lower susceptibility to the BLPs compared to *S. aureus* NTCT8325. Methicillin resistance is only part of the difference between the two strains, but the effect of the acquisition of drug resistance on susceptibility to BLP needs to be investigated in the future.

## Conclusions

BLP has potential for application as an antimicrobial agent in medicine, veterinary science, the food industry, and detergents given its staphylolytic activity, but an efficient production method has not been developed to date. In the present study, we demonstrated the production of the active form of BLP by *B. subtilis*, opening up the possibility of industrial production of BLP. These results are expected to facilitate further applied research on M23A subfamily proteases, including BLP.

## Methods

### Bacterial strains, plasmids, and culture conditions

The bacterial strains and plasmids used in this study are listed in Table 1. *E. coli* DH5α (Nippon Gene, Tokyo, Japan) was used as the host for plasmid preparation and was grown in LB medium Lennox (1% tryptone, 0.5% yeast extract, 0.5% NaCl) supplemented with 100 μg/mL ampicillin. The *B. subtilis* strains were grown in LB medium or modified 2 × L-Mal medium (2% tryptone, 1% yeast extract, 1% NaCl, 7.5% maltose, 7.5 μg/mL MnSO_4_, 6 μg/mL ZnSO_4_·7H_2_O). Where appropriate, antibiotics were added to the media at the following concentrations: 100 μg/mL spectinomycin and 5 μg/mL chloramphenicol. A solid medium was prepared by adding 1.5% agar to LB medium. The protoplast transformation method [37] was used to introduce BLP expression plasmids into *B. subtilis*, and transformants were selected on DM3 medium supplemented with 50 μg/mL tetracycline [37]. For enzyme production, cells were pre-cultured in 1 mL of LB medium supplemented with 15 μg/mL tetracycline at 30 °C for 15 h; an aliquot (100 μL) of the pre-culture broth then was added to 5 mL of modified 2 × L-Mal medium supplemented with 15 μg/mL tetracycline. After further cultivation at 30 °C for 72 h, cells were removed by centrifugation and the culture supernatant was collected.

**Table 1:** Bacterial strains and plasmids used in this study

Strain or plasmid	Relevant genotype or descriptions^a^	Reference or source
Strains		
168	*trpC2*	[^44^]
Dpr9	*trpC2* Δ*epr* Δ*wprA* Δ*mpr* Δ*nprB* Δ*bpr* Δ*nprE* Δ*vpr* Δ*aprE ΔaprX*	This study
Δ*bdbDC*	*trpC2 bdbDC::spc*	This study
Plasmids		
pUC118-Cm^r^Δ*aprX*	Plasmid for markerless deletion of *aprX* gene, containing *cat*	This study
pHY300PLK	Shuttle vector for *E. coli* and *B. subtilis*, containing *amp* and *tet*	Takara Bio
pHY-S237	pHY300PLK carrying *egl-237* with *egl-237* promoter	This study
pHY-S237sBLP	pHY300PLK carrying the signal, pro and mature sequence of *blp* fused to the promoter and signal sequence of *egl-237*	This study
pHY-BLP01	pHY300PLK carrying the signal, pro and mature sequence of *blp* fused to the promoter of *egl-237*	This study
pHY-BLP02	pHY300PLK carrying the pro and mature sequence of *blp* fused to the promoter and signal sequence of *egl-237*	This study
pHY-BLP03	pHY300PLK carrying the mature sequence of *blp* fused to the promoter and signal sequence of *egl-237*	This study

^a^Antibiotic resistance genes are abbreviated in the table as follows: *spc*, spectinomycin; *cat*, chloramphenicol; *amp*, ampicillin; *tet*, tetracycline

### Construction of mutant strains

Primers used in this study are listed in Additional file 2: Table S1. *B. subtilis* strain Dpr9, in which nine genes encoding extracellular proteases (*aprE*, *nprE*, *bpr*, *wprA*, *vpr*, *epr*, *mpr*, *nprB*, and *aprX*) have been deleted from the chromosome, was constructed from Dpr8 [38], in which eight genes encoding extracellular proteases were deleted, by deleting the *aprX* gene using a previously described markerless gene deletion system [38]. Specifically, Fragments 1 and 2 (containing the upstream and downstream regions of *aprX* gene, respectively) were amplified from *B. subtilis* 168 chromosomal DNA with two pairs of primers (primers aprXfw1 and aprXUPr for Fragment 1, primers aprXDNf and aprXrv-repU for Fragment 2). Fragment 3 (containing the *repU* promoter [39] and the chloramphenicol resistance gene) was amplified from a pUC118-CmrΔ*epr* plasmid [38] with primers repUfw and Cmrv1. Fragment 4 was amplified with the primers aprXfw2 and Cmrv2 using a mixture of Fragments 1, 2, and 3 as templates. The resulting amplified fragment was cloned into the SmaI site of pUC118 (Takara Bio, Shiga, Japan) to generate pUC118-CmrΔ*aprX*. pUC118-CmrΔ*aprX* was used for the transformation of Dpr8, and the resulting Δ*aprX* mutant (designated Dpr9) was constructed as described previously [38].

The Δ*bdbDC* mutant, in which the *bdbDC* operon was substituted with a spectinomycin resistance gene (*spc*), was constructed from strain 168 as follows. Fragments 5 and 6 (containing the upstream and downstream regions of the *bdbDC* operon, respectively) were amplified from *B. subtilis* 168 chromosomal DNA with two pairs of primers (primers bdbDCup_fw and bdbDCup(spc)_rv for Fragment 5, primers bdbDCdw(spc)_fw and bdbDCdw_rv for Fragment 6). Fragment 7 (containing the *spc* gene) was amplified with primers spc_fw and spc_rv, using plasmid pDG1727 [40] as a template. Fragment 8 was amplified with primers bdbDCup_fw/bdbDCdw_rv, using a mixture of Fragments 5, 6, and 7 as templates, and then used for the transformation of *B. subtilis* 168. After selection for spectinomycin resistance, proper gene disruption was confirmed by colony PCR.

### Construction of BLP expression plasmids

A BLP coding sequence from *L. enzymogenes* M497-1 (GenBank accession number: BAV99603.1) [25] that was codon-optimized for *B. subtilis* (Additional file 2: Supplementary Methods) was designed, synthesized, and cloned into pUC57 (GenScript Japan, Tokyo, Japan). The resulting plasmid was designated pUC57-BLP. The pHY-S237 plasmid, which was used as a template for pHY-S237sBLP, was constructed as follows. A fragment containing the promoter, coding region, and terminator of the gene encoding the alkaline cellulase Egl-237 (GenBank accession number: BAB19360.1) was amplified from *Bacillus* sp. KSM-S237 chromosomal DNA [41] with primers s237pro_fw and s237ter_rv. Plasmid pHY300PLK was linearized by inverse PCR with primers pHY(s237)_fw and pHY(s237)_rv. Those two fragments were fused using the In-fusion HD cloning kit (Takara Bio), yielding a plasmid designated pHY-S237. The pHY-S237sBLP plasmid, which was used as a template for the BLP expression plasmids, was constructed as follows. A fragment encoding the signal, propeptide, and mature form of BLP was amplified from pUC57-BLP with primers BLP(s237)_fw and BLP(s237)_rv. A vector fragment carrying the promoter, signal sequence, and the terminator of *egl-237* was amplified from pHY-S237 with primers pHYS_fw and pHYS_rv. Those two fragments were fused using the In-fusion HD cloning kit, yielding pHY-S237sBLP. Plasmids used for the expression of wild-type BLP were constructed as follows. PCR fragments were amplified from pHY-S237sBLP with three pairs of primers (primers BLP01_fw and BLP01_rv for pHY-BLP01, primers BLP02_fw and BLP02_rv for pHY-BLP02, and primers BLP03_fw and BLP03_rv for pHY-BLP03) and transformed into *E. coli* to yield plasmids pHY-BLP01, pHY-BLP02, and pHY-BLP03, respectively (Fig. 1). Site-directed mutagenesis at the nucleotide residues corresponding to amino acid 116 of BLP was performed as follows. Forward mutagenic primer Q116X_fw (where X represents the amino acid after the substitution) and common reverse primer Q116_rv were employed for each mutagenesis PCR, using pHY-BLP02 as a template. The resulting PCR fragments were transformed into *E. coli* to generate expression plasmids encoding each mutant protein.

### SDS-PAGE analysis

Sodium dodecyl sulfate–polyacrylamide gel electrophoresis (SDS-PAGE) analysis was performed according to the method of Laemmli [42], as follows. Each culture supernatant or purified protein was mixed with reduced Laemmli sample buffer containing dithiothreitol (DTT; final concentration 200 mM) and incubated at 100 °C for 3 min. Each sample was loaded on an Any kD™ Mini-PROTEAN^®^ TGX Stain-Free™ Protein Gel (Bio-Rad, Hercules, CA, USA) and subjected to electrophoretic separation. Chemifluorescent signals were captured using a Chemi Doc MP Imaging system (Bio-Rad). Precision Plus Protein Unstained Standards (Bio-Rad) were used as molecular weight markers. The protein bands were analyzed using Image Lab software version 4.0 (Bio-rad). The protein level of mature BLP in each culture supernatant was calculated from the intensity of the band at the position of mature BLP (19 kDa), using the serial diluent of purified wild-type BLP (quantified by DC-protein assay kit (Bio-Rad)) as the standard. Non-reducing SDS-PAGE was performed in the same way as above, but using DTT-free Laemmli sample buffer instead of the reduced buffer.

### Purification of BLP variants

For the purification of BLP variants, *B. subtilis* strain 168 harboring pHY-BLP02 or the mutant plasmids was cultivated in modified 2 × L-Mal medium at 30 °C for 72 h. Wild-type and variant BLP enzymes were purified using the same protocol as follows. 2 mL of the culture supernatant was dialyzed overnight against 20 mM Tris–HCl buffer (pH 7.5). The whole volume of the retentate was applied to a Pierce™ Strong Cation Exchange Spin Column, Mini (Thermo Fisher Scientific, Waltham, MA, USA) equilibrated with the same buffer, and the column was washed with 400 µL of 20 mM NaCl in the same buffer. BLP was eluted using 400 µL of 200 mM NaCl in the same buffer, and the buffer was exchanged to 20 mM Tris–HCl (pH 7.5) by ultrafiltration using VIVASPIN 20, MWCO 3,000 (Sartorius AG, Göttingen, Germany). The protein level of wild-type BLP was determined with a DC-protein assay kit (Bio-Rad) using bovine serum albumin as the standard. Protein levels of Gln116 mutants of BLP were determined by SDS-PAGE analysis using wild-type BLP as the standard as follows. An aliquot (500 ng) of each purified protein (quantified by DC-protein assay kit) was applied to SDS-PAGE. The protein levels were calculated from the band intensity of each mutant, using wild-type BLP (quantified by DC-protein assay kit) as the standard. Each variant was electrophoresed in three lanes, and the means of the band intensities of BLP were used for calculations. Finally, the purity of each purified BLP variant was assessed by SDS-PAGE analysis using 500 ng of each purified protein (quantified by SDS-PAGE) per lane (Additional file 1: Fig. S1).

### N-terminal amino acid sequencing

The culture supernatant and the purified BLP were separated by SDS-PAGE and electrotransferred to a PVDF membrane. Bands were stained, excised, and submitted for N-terminal sequencing by Edman degradation (Nippi, Tokyo, Japan).

### Peptide cleavage activity assay

A pentaglycine containing the FRET substrate (D-A2pr(Nma)-Gly-Gly-Gly-Gly-Gly-Lys(Dnp), named FRET-GGGGG) was synthesized by PH Japan Co. (Hiroshima, Japan). FRET-GGGGG contained a highly fluorescent 2-(*N*-methylamino)benzoyl (Nma) group linked to the side chain of the N-terminal d-2,3-diamino propionic acid (d-A2pr) residue. This group is efficiently quenched by a 2,4-dinitrophenyl (Dnp) group linked to the side chain of the C-terminal Lys residue.

Culture supernatants and purified BLP variants were diluted in 200 µL of assay buffer (20 mM Tris–HCl (pH 7.5)) in a 96-well black plate. Then, 10 µL of 1 mM FRET-GGGGG in the assay buffer was added to each well. Fluorescence was measured once per minute at excitation/emission wavelengths of 340/440 nm (respectively) at 30 °C in a Tecan Infinite M200 plate reader (Tecan, Männedorf, Switzerland). An aliquot of 200 µL of an equimolar mixture of FRETS-25-STD1 (Peptide Institute, Inc. Osaka, Japan) and FRETS-25-STD2 (Peptide Institute, Inc.) in the assay buffer was used as the standard. FRETS-25-STD1 (d-A2pr(Nma)-Gly) contains Nma, which acts as a fluorophore in the FRET-GGGGG. FRETS-25-STD2 (Ala-Phe-Pro-Lys(Dnp)-d-Arg-d-Arg) contains Dnp, which acts as a quencher in the FRET-GGGGG.

One unit (U) of the pentaglycine cleavage activity was defined as the amount of enzyme needed to exhibit a change in fluorescence intensity equivalent to 1 nmol of FRETS-25-STD1 and 1 nmol of FRETS-25-STD2 per minute.

### Staphylolytic activity assay

*S. aureus* NCTC8325 was obtained from the National Collection of Type Cultures. *S. aureus* NCTC8325 was cultured in 10 mL of Soybean-Casein Digest Broth “DAIGO” (FUJIFILM Wako Pure Chemical Corp., Osaka, Japan) at 37 °C for 24 h, and the cells were collected by centrifugation. The cells were suspended in 10 mL of assay buffer (20 mM Tris–HCl (pH 7.5)), preincubated at 30 °C for 10 min, and diluted with the assay buffer to yield an absorbance of 1.0 at 600 nm. The reaction was initiated by adding 4 µL of 33 µg/mL enzyme to 100 µL of the cell suspension dispensed into a 96-well plate. After incubation at 30 °C for 5 min, the absorbance at 600 nm was measured using a Tecan Infinite M200 plate reader. The measurements were corrected to a 1-cm pathlength by dividing by the measurement of 104 µL of the cell suspension with an absorbance of 1.0 at 600 nm with a 1-cm pathlength. One lytic unit (LU) was defined as the amount of enzyme needed to decrease the absorbance at 600 nm by 0.01 compared to the blank.

### Bactericidal assay

Methicillin-resistant *S. aureus* (MRSA) ATCC43300 was obtained from the American Type Culture Collection. *S. aureus* NCTC8325 and *S. aureus* ATCC43300 were cultured (separately) in 2 mL of Soybean-Casein Digest Broth “DAIGO” at 37 °C for 24 h, and the cells were collected by centrifugation. The cells then were suspended in an appropriate volume of assay buffer (20 mM Tris–HCl (pH 7.5)) to yield an absorbance of 0.15 at 600 nm. An aliquot (10 µL) of the cell suspension was added to 200 µL of enzyme solution diluted in assay buffer and mixed well. After incubation at 30 °C for 60 min, the cells were diluted tenfold and 1000-fold with Diluent with Lecithin & Polysorbate 80 “DAIGO” (FUJIFILM Wako Pure Chemical Corp.). An aliquot (100 µL) of each dilution was plated on Soybean-Casein Digest Agar “DAIGO” (FUJIFILM Wako Pure Chemical Corp.) plates and incubated at 37 °C for 24 h.

The number of colonies formed was counted for each plate. The MBC was the lowest concentration of the enzyme that caused a reduction in colony count of at least 3 logs compared with the initial bacterial concentration.

### Homology modeling

The protein sequence of BLP from *L. enzymogenes* M497-1 was retrieved from the GenBank database as Accession Number BAV99603.1 (377 amino acid residues). The mature sequence of BLP, consisting of residues 199 to 377, was used for the structure prediction. The structural model of mature BLP was predicted by Discovery Studio 2017 R2 (Dassault Systèmes, Vélizy-Villacoublay, France) using as a template the crystal structure of LasA protease (PDB code 3IT5) [43], which shares 43% amino acid identity with mature BLP.

## Supplementary Information

**Additional file 1: Fig. S1** SDS-PAGE analysis of purified BLP variants

**Additional file 2: Table S1**: Primers used in this study. **Supplementary Methods**.

## Data Availability

All data generated or analyzed during this study are included in this published article and its Additional file.

## Abbreviations

BLP: Beta-lytic protease
ORF: Open reading frame
MSSA: Methicillin-susceptible *Staphylococcus aureus*
MRSA: Methicillin-resistant *Staphylococcus aureus*
MBC: Minimum bactericidal concentration
PDB: Protein data bank
SDS-PAGE: Sodium dodecyl sulfate–polyacrylamide gel electrophoresis
FRET: Förster (Fluorescence) resonance energy transfer
DTT: Dithiothreitol
