# Supplementary figures and images for: Heterologous production of active form of beta-lytic protease by Bacillus subtilis and improvement of staphylolytic activity by protein engineering
Source: Microb Cell Fact. 2021 Dec 28;20:231. doi: 10.1186/s12934-021-01724-x (PMC8715609; doi:10.1186/s12934-021-01724-x)

Figure S1 T. Hioki *et al.*

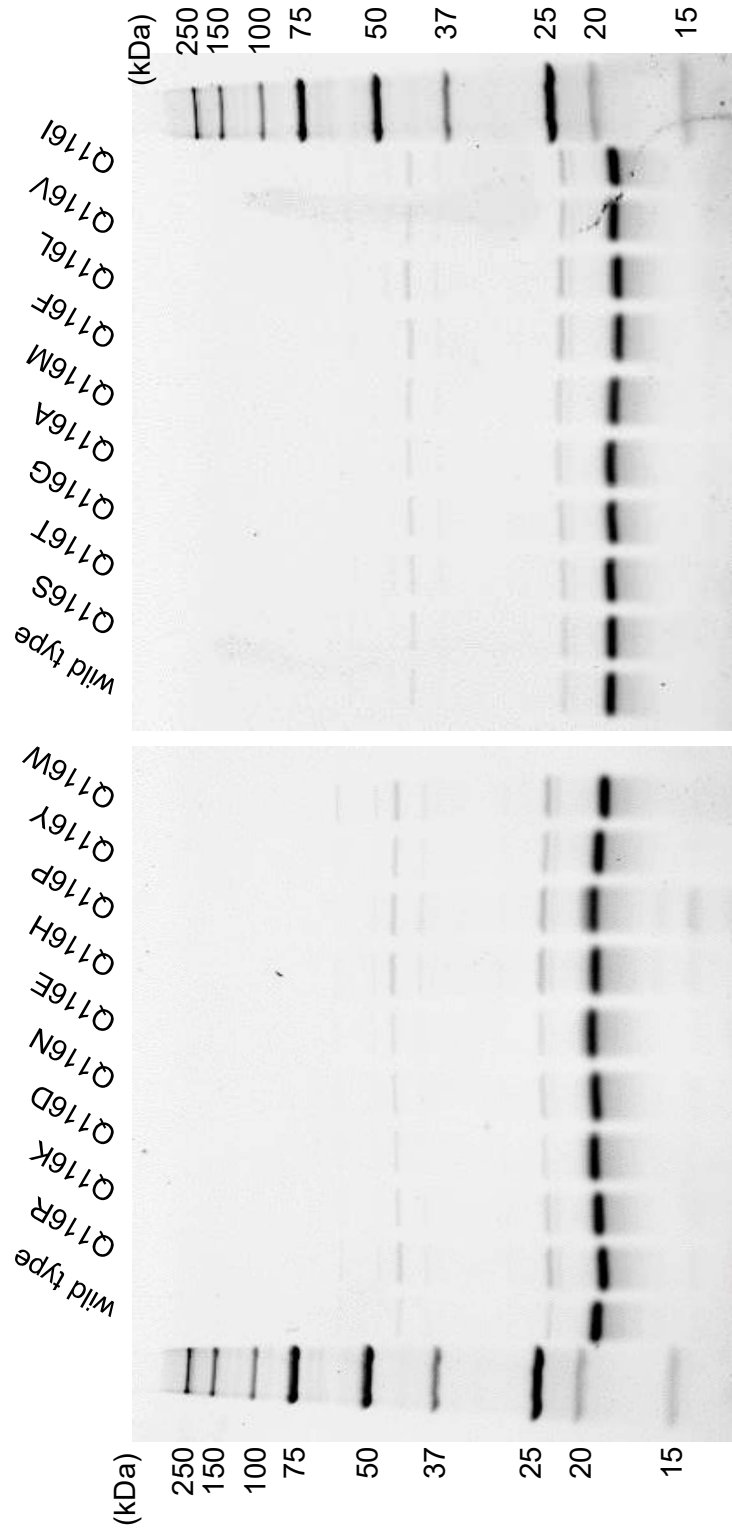

Supplement: Supplementary file 1 — Additional file 1: Fig. S1 SDS-PAGE analysis of purified BLP variants [file 12934_2021_1724_MOESM1_ESM.pdf]
