# Supplementary material for: Heterologous production of active form of beta-lytic protease by Bacillus subtilis and improvement of staphylolytic activity by protein engineering
Source: Microb Cell Fact. 2021 Dec 28;20:231. doi: 10.1186/s12934-021-01724-x (PMC8715609; doi:10.1186/s12934-021-01724-x)
Supplement: Supplementary file 2 — Additional file 2: Table S1: Primers used in this study. Supplementary Methods. [file 12934_2021_1724_MOESM2_ESM.docx]

# Additional files

## Figure S1: SDS-PAGE analysis of purified BLP variants

500 ng of each purified protein (quantified by SDS-PAGE) was applied to SDS-PAGE.

## Table S1: Primers used in this study

| **Primer** | **Sequence (5'→3')** |
| --- | --- |
| aprXfw1 | caacctcacttggcaaataaatgt |
| aprXUPr | tttctgcgttcaccatttgtaccatagag |
| aprXDNf | acaaatggtgaacgcagaaaattccgttc |
| aprXrv-repU | ctgatctcgacttcgttcatagatcggcgcctgaaa |
| repUfw | gaacgaagtcgagatcag |
| Cmrv1 | gctgtaatataaaaaccttct |
| aprXfw2 | atataggtttaaggaggg |
| Cmrv2 | aactaacggggcaggtta |
| bdbDCup_fw | atggaactgagatccttgctg |
| bdbDCup(spc)_rv | attcacgaacgaaaatcgataccacaaccgtaaggatgac |
| bdbDCdw(spc)_fw | attttagaaaacaataaacccattaagccatgtgtcagcg |
| bdbDCdw_rv | tccgctttcagcacaagcg |
| spc_fw | atcgattttcgttcgtgaatacatg |
| spc_rv | ggtttattgttttctaaaatctgattaccaattagaatg |
| s237pro_fw | caacaggcttatatttagaggaa |
| s237ter_rv | tccagttatgcaagaaaaaggttatctaaaag |
| pHY(s237)_fw | tcttgcataactggatccgtcgacctgcag |
| pHY(s237)_rv | aatataagcctgttggatccccgggaattcctg |
| BLP(s237)_fw | gaaggaaacactcgtatgaaaaaaatctcaaaagc |
| BLP(s237)_rv | aactagtttaatagattagttcggtccaggattcac |
| pHYS_fw | tctattaaactagttatagggttatctaaagg |
| pHYS_rv | acgagtgtttccttctgctgc |
| BLP01_fw | ttaggaggtaatatgatgaaaaaaatctcaaaagctggtctgg |
| BLP01_rv | catattacctcctaaatatttttaaagtaattgaatc |
| BLP02_fw | tgcagcatctgctcagggacatggattaa |
| BLP02_rv | tgagcagatgctgcaagagctgccggaa |
| BLP03_fw | ttgcagcatctccgaatggactgcttca |
| BLP03_rv | tcggagatgctgcaagagctgccggaa |
| Q116R_fw | ctgtgcaacggaggcagaagcacaggaccgcatgaa |
| Q116K_fw | ctgtgcaacggaggcaaaagcacaggaccgcatgaa |
| Q116D_fw | ctgtgcaacggaggcgatagcacaggaccgcatgaa |
| Q116N_fw | ctgtgcaacggaggcaacagcacaggaccgcatgaa |
| Q116E_fw | ctgtgcaacggaggcgaaagcacaggaccgcatgaa |
| Q116H_fw | ctgtgcaacggaggccatagcacaggaccgcatgaa |
| Q116P_fw | ctgtgcaacggaggcccgagcacaggaccgcatgaa |
| Q116Y_fw | ctgtgcaacggaggctacagcacaggaccgcatgaa |
| Q116W_fw | ctgtgcaacggaggctggagcacaggaccgcatgaa |
| Q116S_fw | ctgtgcaacggaggctctagcacaggaccgcatgaa |
| Q116T_fw | ctgtgcaacggaggcacaagcacaggaccgcatgaa |
| Q116G_fw | ctgtgcaacggaggcggaagcacaggaccgcatgaa |
| Q116A_fw | ctgtgcaacggaggcgcaagcacaggaccgcatgaa |
| Q116M_fw | ctgtgcaacggaggcatgagcacaggaccgcatgaa |
| Q116C_fw | ctgtgcaacggaggctgcagcacaggaccgcatgaa |
| Q116F_fw | ctgtgcaacggaggctttagcacaggaccgcatgaa |
| Q116L_fw | ctgtgcaacggaggcttaagcacaggaccgcatgaa |
| Q116V_fw | ctgtgcaacggaggcgttagcacaggaccgcatgaa |
| Q116I_fw | ctgtgcaacggaggcatcagcacaggaccgcatgaa |
| Q116_rv | gcctccgttgcacagagcctgcgc |

## Supplementary Methods

**Codon-optimized nucleotide sequence of *blp* for expression in *B. subtilis***

ATGAAAAAAATCTCAAAAGCTGGTCTGGGACTGGCTCTGGTCTGTGCTCTGGCGACGATTGGAGGCAACGCATCTGCTCAGGGACATGGATTAAGCGGCGAAGATCTGGTTTACTCTTACGATGAAATGTTTGATTTTGATATCGATGCCTACCTGGCAAAACATGCGCCGCATCTGCATAAACATAGCGAAGAAATCTCTCATTGGGCCGGATATTCTGGCATTTCACCGAAAGTTCTTATCGCATTAATGGAACAACAGTCAGGAGCTGTGAGCGCCAAAAGAGCAACAAATCGCCCGTTTGGCAAACTTGCCAGAGCAGATGGATTTGGCGCCCAAACACGCGAAGTGGCGTTAGCTCTGAGAGAATCTCTTTATGAACGCGATCCGGATGGAGCCAAAGGCCCGGTCACATTAGCCAGAGCAAACCCGCTGCAGGCACTTTTTGAACGCTCAGGAGATAATGAACCGGCAGCGGCTTTAAGAGGAGATGGCGAATTTCAACTTGTCTACGGCAGATTATTTAACGAACCGCGCCAGGCAAAAGCCGCAAGCGATAGATTTGCGAAAGCTGGACCGGATGTTCAACCGTTATCTCCGAATGGACTGCTTCAGTTTCCGTTTCCGAGAGGCGCATCTTGGCATGTGGGCGGAGCTCATACAAACACAGGATCAGGCAATTATCCGATGTCAAGCCTGGATATGTCAAGAGGCGGAGGCTGGGGAAGCAATCAAAACGGCAATTGGGTTTCAGCGAGCGCGGCTGGATCTTTTAAACGCCATTCTTCATGCTTTGCTGAAATTGTTCATACAGGCGGCTGGTCAACAACATACTACCATCTGATGAACATCCAGTACAATACAGGCGCGAACGTTAGCATGAATACAGCCATCGCAAACCCGGCTAATACACAAGCGCAGGCTCTGTGCAACGGAGGCCAAAGCACAGGACCGCATGAACATTGGTCACTGAAACAGAACGGCTCATTTTACCATCTGAACGGAACATACCTTTCAGGCTATAGAATCACAGCGACAGGCAGCTCTTATGATACAAATTGTAGCCGCTTTTATTTGACAAAAAATGGACAGAACTACTGCTATGGTTATTATGTGAATCCTGGACCGAACTAA

**Predicted amino acid sequence of BLP expressed in *B. subtilis*.**

The signal sequences are underlined with dotted lines, and the pro sequences are underlined with straight lines.

>pHY-BLP01

MKKISKAGLGLALVCALATIGGNASAQGHGLSGEDLVYSYDEMFDFDIDAYLAKHAPHLHKHSEEISHWAGYSGISPKVLIALMEQQSGAVSAKRATNRPFGKLARADGFGAQTREVALALRESLYERDPDGAKGPVTLARANPLQALFERSGDNEPAAALRGDGEFQLVYGRLFNEPRQAKAASDRFAKAGPDVQPLSPNGLLQFPFPRGASWHVGGAHTNTGSGNYPMSSLDMSRGGGWGSNQNGNWVSASAAGSFKRHSSCFAEIVHTGGWSTTYYHLMNIQYNTGANVSMNTAIANPANTQAQALCNGGQSTGPHEHWSLKQNGSFYHLNGTYLSGYRITATGSSYDTNCSRFYLTKNGQNYCYGYYVNPGPN

>pHY-BLP02

MLRKKTKQLISSILILVLLLSLFPAALAASAQGHGLSGEDLVYSYDEMFDFDIDAYLAKHAPHLHKHSEEISHWAGYSGISPKVLIALMEQQSGAVSAKRATNRPFGKLARADGFGAQTREVALALRESLYERDPDGAKGPVTLARANPLQALFERSGDNEPAAALRGDGEFQLVYGRLFNEPRQAKAASDRFAKAGPDVQPLSPNGLLQFPFPRGASWHVGGAHTNTGSGNYPMSSLDMSRGGGWGSNQNGNWVSASAAGSFKRHSSCFAEIVHTGGWSTTYYHLMNIQYNTGANVSMNTAIANPANTQAQALCNGGQSTGPHEHWSLKQNGSFYHLNGTYLSGYRITATGSSYDTNCSRFYLTKNGQNYCYGYYVNPGPN

>pHY-BLP03

MLRKKTKQLISSILILVLLLSLFPAALAASPNGLLQFPFPRGASWHVGGAHTNTGSGNYPMSSLDMSRGGGWGSNQNGNWVSASAAGSFKRHSSCFAEIVHTGGWSTTYYHLMNIQYNTGANVSMNTAIANPANTQAQALCNGGQSTGPHEHWSLKQNGSFYHLNGTYLSGYRITATGSSYDTNCSRFYLTKNGQNYCYGYYVNPGPN
